# RNA-Binding Proteins and Alternative Splicing Genes Are Coregulated in Human Retinal Endothelial Cells Treated with High Glucose

**DOI:** 10.1155/2022/7680513

**Published:** 2022-03-09

**Authors:** Hongran Zhao, Hui Kong, Bozhao Wang, Sihui Wu, Tianran Chen, Yan Cui

**Affiliations:** ^1^Department of Ophthalmology, Qilu Hospital of Shandong University, Shandong University, Jinan, Shandong Province, China; ^2^School of Medicine, Shandong University, Jinan, Shandong Province, China; ^3^Shandong University of Traditional Chinese Medicine, Jinan, Shandong Province, China

## Abstract

To explore the relevant RNA-binding proteins (RBPs) and alternative splicing events (ASEs) in diabetic retinopathy (DR). We devised a comprehensive work to integrate analyses of the differentially expressed genes, including differential RBPs, and variable splicing characteristics related to DR in human retinal endothelial cells induced by low glucose and high glucose in dataset GSE117238. A total of 2320 differentially expressed genes (DEGs) were identified, including 1228 upregulated genes and 1092 downregulated genes. Further analysis screened out 232 RBP genes, and 42 AS genes overlapped DEGs. We selected high expression and consistency six RBP genes (FUS, HNRNPA2B1, CANX, EIF1, CALR, and POLR2A) for coexpression analysis. Through analysis, we found eight RASGs (MDM2, GOLGA2P7, NFE2L1, KDM4A, FAM111A, CIRBP, IDH1, and MCM7) that could be regulated by RBP. The coexpression network was conducted to further elucidate the regulatory and interaction relationship between RBPs and AS. Apoptotic progress, protein phosphorylation, and NF-kappaB cascade revealed by the functional enrichment analysis of RASGs regulated by RBPs were closely related to diabetic retinopathy. Furthermore, the expression of differentially expressed RBPs was validated by qRT-PCR in mouse retinal microvascular endothelial cells and retinas from the streptozotocin mouse model. The results showed that *Fus*, *Hnrnpa2b1*, *Canx*, *Calr*, and *Polr2a* were remarkedly difference in high-glucose-treated retinal microvascular endothelial cells and *Fus*, *Hnrnpa2b1*, *Canx*, and *Calr* were remarkedly difference in retinas from streptozotocin-induced diabetic mice compared to control. The regulatory network between identified RBPs and RASGs suggests the presence of several signaling pathways possibly involved in the pathogenesis of DR. The verified RBPs should be further addressed by future studies investigating associations between RBPs and the downstream of AS, as they could serve as potential biomarkers and targets for DR.

## 1. Introduction

Diabetic retinopathy (DR) is a common and specific microvascular complication of diabetes mellitus and a leading cause of vision impairment and blindness in the working-age population [1]. It has been estimated that 693 million people will have diabetes by 2045, 35% of whom will have DR. Severe degrees of DR have been associated with impaired quality of life, reduced physical, emotional, and social well-being, as well as a serious family burden [2]. The current treatments for DR are applicable only at advanced stages and often associated with poor prognosis, while early diagnosis and preventative measures are of great importance to prevent DR. Therefore, further understanding of the key mechanism that could lead to early recognition of DR is vital for preventing the degradation of visual acuity and developing new, more effective treatments.

Recent studies have suggested that genetic and epigenetic, such as dysfunction in noncoding RNA (circRNA [3], lncRNA [4], miRNA [5], siRNA [6]), m6A mRNA methylation [7], and alternative splicing have an important role in the occurrence and progression of DR. Alternative splicing (AS) is a crucial role in various biological and pathological processes. In humans, high-throughput genome-wide analyses have revealed that about 95% of multiexon genes undergo AS [8]. Through AS, hundreds of thousands of RNA isoforms with distinct structural properties, localization patterns, and translation efficiencies can be expressed as protein isoforms with diverse functions [9]. Moreover, the misregulation of AS has been linked to various diseases, such as central nervous system diseases [10], heart diseases [11], carcinoma [12], immune diseases [13], and metabolic diseases [14]. Preexisting studies have also revealed the alternative splicing of VEGF [15], TUBD1 [16] in DR. However, most AS variants as well as the underlying regulatory mechanisms responsible for the pathogenesis of DR remain unclear.

RNA-binding proteins (RBPs) are vital regulators of an mRNA life cycle, including their transcription, splicing export, and transport to degradation, deadenylation, storage, silencing, and mRNA translation (protein synthesis) [17, 18]. Along with the spliceosome complex, RBPs have a major role in creating cell-type-specific regulation of alternative splicing [10, 19, 20]. Previous researchers have revealed the regulatory role of zinc finger RNA-binding protein [21] and the ELAVL1 (or human antigen R (HuR)) RNA-binding protein [22] in DR. However, so far, no studies investigate RBPs in regulating alternative splicing events (ASEs) during the progression of DR.

In this study, we used bioinformatics methods and expression analysis techniques to explore the relationship between RBPs and AS in DR. Bioinformatics analysis technology was employed to comprehensively and deeply analyze the differentially expressed genes (DEGs) in the dataset GSE117238 and then explore the differentially expressed RBPs and regulated alternative splicing genes (RASGs) in this dataset. To further clarify the role of ASEs, the regulatory network between ASEs and RBPs was analyzed. We found that FUS, HNRNPA2B1, CANX, EIF1, CALR, and POLR2A are potential targets, through which these RBPs modulate downstream AS during DR progression and provide valuable clues for further validation.

## 2. Materials and Methods

### 2.1. Retrieval and Process of Public Data

Data GSE117238 was downloaded from the Sequence Read Archive (SRA). SRA Run files were converted to fastq format with NCBI SRA Tool fastq-dump [23]. The raw reads were trimmed of low-quality bases using a FASTX-Toolkit (v.0.0.13; http://hannonlab.cshl.edu/fastx_toolkit/). Then, the clean reads were evaluated using FastQC (http://www.bioinformatics.babraham.ac.uk/projects/fastqc) [24].

### 2.2. Read Alignment and DEG Analysis

Clean reads were aligned to the human GRch38 genome by tophat2 [25], allowing 4 mismatches. Uniquely mapped reads were ultimately applied to calculate read number and reads per kilobase of exon per million fragments mapped (RPKM) for each gene. The expression levels of genes were evaluated using RPKM. The software edgeR [26], which is specifically designed to analyze the differential expression of genes, was applied to screen the RNA-Seq data for DEGs [24]. The results were analyzed based on the fold change (FC ≥ 2 or ≤0.5) and false discovery rate (FDR ≤ 0.05) to assess whether a gene was differentially expressed.

### 2.3. Alternative Splicing Analysis

The ASEs and regulated alternative splicing events (RASEs) between the samples were defined and quantified using the ABLas pipeline as described previously [27, 28]. In brief, ABLas detection of ten types of ASEs was based on the splice junction reads, including exon skipping (ES), alternative 5′ splice site (A5SS), alternative 3′ splice site (A3SS), intron retention (IR), mutually exclusive exons (MXE), mutually exclusive 5′ UTRs (5pMXE), mutually exclusive 3′ UTRs (3pMXE), cassette exon, A3SS&ES, and A5SS&ES [29].

For sample pair comparison, Fisher's exact test was selected to determine statistical significance, using the samples' alternative reads and model reads as input data. We calculated the changed ratio of alternatively spliced reads and constitutively spliced reads between compared samples, which was defined as the RASE ratio. The RASE ratio ≥ 0.2 and *p* value ≤ 0.05 were set as the threshold for RASE detection. For repetition comparison, Student's *t*-test was used to evaluate the significance of the ratio alteration of AS events. Those events, which were significant at *p* value cutoff of 0.05, were considered RASEs [30].

### 2.4. Coexpression Analysis

To investigate the regulatory mode between RBP expression and alternative splicing (percent-splice-in, PSI), we calculated Pearson's correlation coefficients (PCCs) between them and classified their relation into three classes: positive correlated, negative correlated, and noncorrelated based on the PCC value [23].

### 2.5. Functional Enrichment Analysis

Gene Ontology (GO) terms and Kyoto Encyclopedia of Genes and Genomes (KEGG) pathways were identified using the KOBAS 2.0 server to analyze the biological process and molecular function of DEGs, ASEs, and RBPs. The Benjamini-Hochberg FDR controlling procedure and the hypergeometric test were used to define the enrichment of each term. Reactome^2^ pathway profiling was also applied to the functional enrichment analysis of the sets of selected genes. A *p* value < 0.005 was regarded as the cutoff criterion [23].

### 2.6. Cell Culture and HG Treatment

The mouse retinal microvascular endothelial cell line, mRMVECs, was purchased from the Qingqi company (Shanghai, China) and cultured in 6-well plates containing DMEM (Gibco, Waltham, MA) supplemented with 10% FBS (Gibco, Waltham, MA) in a humidified atmosphere containing 5%CO_2_/95% air at 37°C. For passaging, cells were digested with trypsin–EDTA solution. mRMVECs were diluted to 2 × 10^5^/mL and incubated with low glucose media until confluent. After washing with PBS, the cells were further cultured for 48 h in 5.5 mM glucose (low glucose, LG) and 25 mM glucose (high glucose, HG) media.

### 2.7. Diabetic Mouse Models Induced with Streptozotocin (STZ)

To establish models of diabetes in mice, 50 mg/kg of STZ, freshly dissolved in citrate buffer (50 mM, pH 4.5), was intraperitoneally injected on a daily basis for 5 d into 8-week-old male C57BL/6J mice (Vital River Laboratories, Beijing, China); the same volume of citrate buffer without STZ was used for a control group. The model was considered successfully established if blood glucose > 16.7 mmol/L. After 8 weeks, the eyes were enucleated, and the retinal tissues were isolated.

All the animals were housed in an environment with a temperature of 22 ± 1°C, relative humidity of 50 ± 1%, and a light/dark cycle of 12/12 hr. All animal studies (including the mouse euthanasia procedure) were done in compliance with Qilu Hospital of Shandong University institutional animal care regulations and ARVO animal statement. Application format for ethical approval for research involving animals was approved on December 22, 2020, and the approval number is Dwll-2020-028.

### 2.8. qRT-PCR

Total RNA was extracted from mRMVECs and retinal specimens using TRIzol reagent (Invitrogen, Waltham, MA) and reverse transcribed into complementary DNA using the ReverTra Ace qPCR RT Kit (TOYOBO, Osaka, Japan), following the manufacturer's introductions. The PCR was conducted by a PCR instrument and SYBR® Green Realtime PCR Master Mix (TOYOBO, Osaka, Japan); the sequence of the primers is listed in Table 1. The expression of detected genes was standardized to GAPDH and analyzed by the 2-*ΔΔ*Ct method.

### 2.9. Statistical Analysis

Principal component analysis (PCA) was performed by R package factoextra (https://cloud.r-project.org/package=factoextra) to show the clustering of samples with the first two components. After normalizing the reads by TPM (Tags Per Million) of each gene in samples, in-house-script (sogen) was used to visualize next-generation sequence data and genomic annotations. The pheatmap package (https://cran.r-project.org/web/packages/pheatmap/index.html) in R was used to perform the clustering based on Euclidean distance. Student's *t*-test was used for comparisons between two groups [23]. Globaltest [31, 32] was used to test the association of the normalized gene expression level of RBPs in different samples with a different phenotype. The data of mRMVECs and mouse experiments are presented as the mean ± SD. Differences among experimental groups were analyzed by a 2-tailed Student's *t*-test using GraphPad Prism 8.0 software (GraphPad, San Diego, CA). A *p* value < 0.05 was considered to be statistically significant.

## 3. Results

### 3.1. Genes Are Differentially Expressed in Human Retinal Endothelial Cells (HRECs) Treated with Low Glucose (LG) and High Glucose (HG)

**Figure 1:**
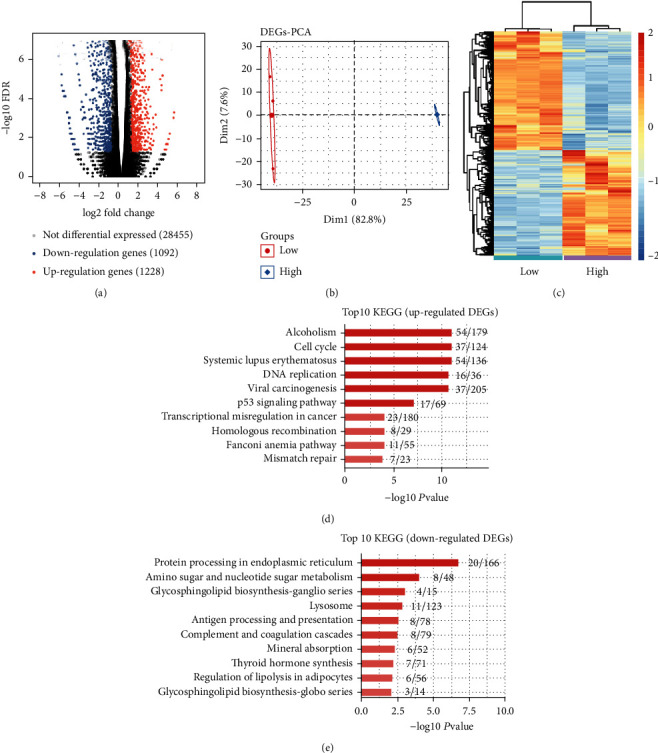
Transcriptome analysis of differentially expressed genes (DEGs) in human retinal endothelial cells (HRECs) treated with hypoglycemia (LG) and hyperglycemia (HG). (a) Volcano gram showing all DEGs between LG and HG samples. *Q* value ≤ 0.05, FC (fold change) ≥ 2 or ≤0.5. (b) Principal component analysis (PCA) of LG and HG in HRECs based on FPKM value of all DEGs. The ellipse for each group is the confidence ellipse. (c) Heatmap for 3 LG and 3 HG samples, based on all DEG FPKM values. (d, e) The KEGG analysis of DEGs, dividing into up- and downregulated genes.

Differentially expressed genes (DEGs) identified from the transcription profile dataset GSE117238 are shown in the volcano gram (Figure 1(a)). A total of 2320 DEGs in HRECs treated with LG and HG were identified, including 1228 upregulated genes and 1092 downregulated genes. Through principal component analysis (PCA), the groups were clearly clustered in distinct regions of the PCA graph (Figure 1(b)). Supervised hierarchical clustering and heatmap were constructed to show DEG expression in 3 LG and 3 HG samples (Figure 1(c)). The GO term enrichment analysis showed that the top ten pathways enriched in upregulated DEGs were “alcoholism, cell cycle, systemic lupus erythematosus, DNA replication, viral carcinogenesis, p53 signaling pathway, transcriptional misregulation in cancer, homologous recombination, Fanconi anemia pathway, and mismatch repair” (Figure 1(d)), while “protein processing in the endoplasmic reticulum, amino sugar and nucleotide sugar metabolism, glycosphingolipid, biosynthesis–ganglio series, lysosome, antigen processing and presentation, complement and coagulation cascades, mineral absorption, thyroid hormone synthesis, regulation of lipolysis in adipocytes, and glycosphingolipid biosynthesis–globo series” were enriched in downregulated DEGs (Figure 1(e)). The GO terms (Supplementary Figure S1A, B) and Reactome analysis (Supplementary Figure S1C, D) were further performed, and the upregulated DEGs were significantly enriched in DNA replication, mitosis, cell cycle, and so on. The downregulated DEGs were significantly enriched in the endoplasmic reticulum, unfolded protein response, unfolded protein responses, and so on.

### 3.2. Different Doses of Glucose-Regulated Alternative Splicing Events (RASEs) and Genes in HRECs

**Figure 2:**
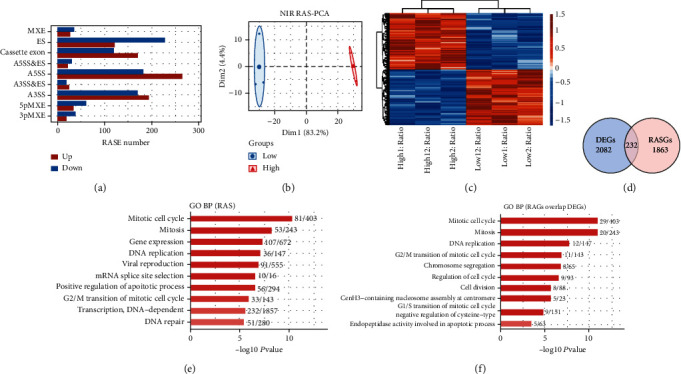
Transcriptome analysis of alternative splicing events (ASEs) in human retinal endothelial cells (HRECs) treated with hypoglycemia (LG) and hyperglycemia (HG). (a) Classification of all the regulated alternative splicing events (RASEs). *X*-axis: RASE number. (b) Principal component analysis (PCA) of LG and HG in HRECs based on PSI value of all different nonintron retention (NIR). The ellipse for each group is the confidence ellipse. (c) PSI heatmap of all NIR RASEs among HG samples compared to LG samples. AS filtered should have detectable splice junctions in all samples and at least 80% of samples should have ≥10 splice junction reads supporting the AS. (d) Venn diagram of DEGs and regulated alternative splicing genes (RASGs); *p* value = 1. (e) The GO analysis of RASGs in HG samples compared to LG samples. (f) The GO analysis of genes overlapped by DEGs and RASGs.

The classification of all the ASEs is shown in Supplementary Figure S2A. Different 2853 ASEs were found between LG- and HG-treated HRECs; A5SS, A3SS, and ES were the most frequent ASEs (Figure 2(a)). Due to the high false-positive rate of IR (intron retention), we chose nonintron retention (NIR) for the next analysis. The PCA of LG samples and HG samples in all different NIR is shown in Figure 2(b). There was a clear spatial separation between the samples. PSI heatmap was constructed to show all NIR RASEs among HG samples compared to LG samples (Figure 2(c)).

The GO analysis showed that the RASGs in HG samples were mainly enriched in “mitotic cell cycle, mitosis, gene expression, DNA replication, viral reproduction, mRNA splice site selection, positive regulation of the apoptotic process, G2/M transition of the mitotic cell cycle, transcription, DNA-dependent, and DNA repair” compared to LG samples (Figure 2(e)). Furthermore, the KEGG analysis showed that the RASGs between LG samples and HG samples were mainly enriched in “cell cycle, apoptosis, spliceosome, apoptosis–multiple species, neurotrophin signaling pathway, shigellosis, insulin resistance, insulin signaling pathway, pathogenic *Escherichia coli* infection, and p53 signaling pathway” (Supplementary Figure S2B).

The Venn diagram further revealed 232 genes intersected between DEGs and RASGs (Figure 2(d)). The GO analysis showed that genes overlapped by DEGs and RASGs were mainly enriched in “mitotic cell cycle, mitosis, DNA replication, G2/M transition of the mitotic cell cycle, chromosome segregation, regulation of cell cycle, cell division, CenH3-containing nucleosome assembly at the centromere, G1/S transition of mitotic cell cycle, and negative regulation of cysteine-type endopeptidase activity involved in the apoptotic process” (Figure 2(f)). In addition, the KEGG analysis revealed that genes overlapped by DEGs and RASGs were mainly enriched in “cell cycle, p53 signaling pathway, apoptosis, pathogenic *Escherichia coli* infection, DNA replication, amino sugar and nucleotide sugar metabolism, herpes simplex infection progesterone-mediated oocyte maturation, and phagosome and pyrimidine metabolism” (Supplementary Figure S2C).

### 3.3. Differentially Expressed RBPs Are Coexpressed with the Alternative Splicing Genes

**Figure 3:**
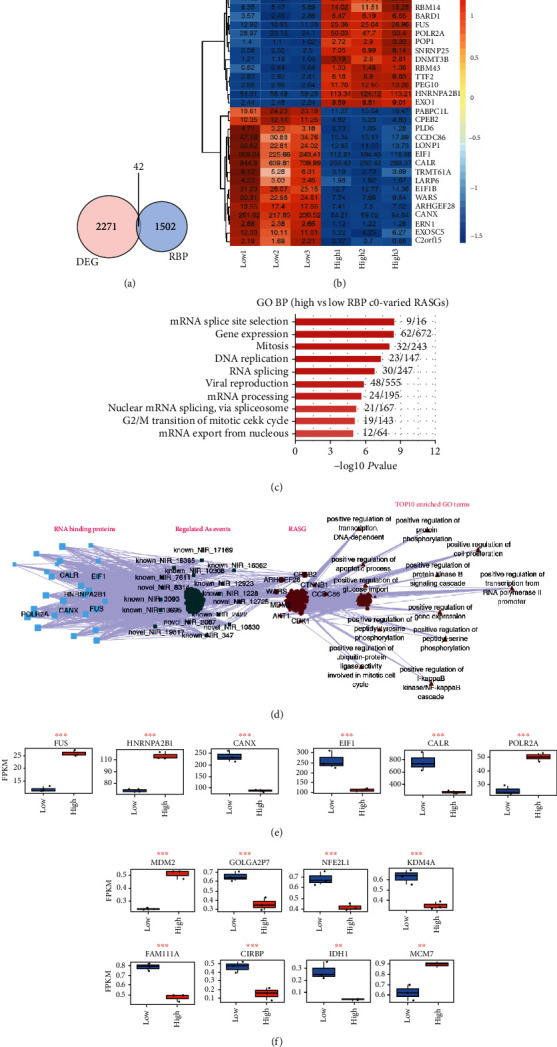
Network between differential RNA-binding proteins (RBPs) and regulated alternative splicing genes (RASGs) in hypoglycemia and hyperglycemia samples. (a) Venn diagram showing differentially expressed RNA-binding protein genes; *p* value = 1. (b) Coexpression analysis was carried out according to overlap gene and RAS of non-intron retention (NIR), and then, 80% of samples were screened for the Parameter: value ≤ 0.01. (c) The GO analysis of RASGs regulated by RBPs. (d) The coderegulation of alternative splicing network between RBPs (the leftmost part) and RASEs (the middle left part, which only includes NIR RASEs). The top enriched GO terms of codisturbed RASGs (the middle right part) were shown in brown (the rightmost part). (e) The boxplot showing the RBPs marked in (d). (f) The boxplot showing the RASGs. ^∗∗∗^*p* < 0.001 and ^∗∗^*p* < 0.01.

In the Venn diagram, 43 intersecting genes related to DEGs and RBPs were found in LG- and HG-treated HRECs (Figure 3(a)). Through the above analysis, significant differences in the expression of DEGs and RASGs were found between the LG and HG samples. Thus, we conducted a coexpression analysis of RPBs and RASGs. As many RBP genes demonstrated influence on ASEs, we performed clustering analysis of RBP-regulated ASGs (Figure 3(b)).

In order to elucidate the molecular implication of RBPs on ASEs, the relation of RASGs with DR-related pathways is analyzed and visualized in Figure 3(c) and Supplementary Figure S3B. The GO analysis was further conducted to show the RASGs regulated by RBPs were mainly enriched in “mRNA splicing site selection, gene expression, mitosis, DNA replication, RNA splicing, viral reproduction, mRNA processing, nuclear mRNA splicing, via spliceosome, G2/M transition of mitotic cell cycle, and mRNA export from nucleus” (Figure 3(c)). The KEGG analysis indicated that RBP-regulated ASEs between two doses of glucose-treated HRECs were mainly enriched in “cell cycle, spliceosome, pathogenic Escherichia coli infection, p53 signaling pathway, insulin signaling pathway, mTOR signaling pathway, bacterial invasion of epithelial cells, ubiquitin-mediated proteolysis, insulin resistance, and apoptosis–multiple species” (Supplementary Figure S3A). Moreover, the Reactome analysis of RBP-regulated ASEs was mainly enriched in “gene and protein expression by JAK-STAT signaling alter interleukin-12 stimulation, regulation of TP53 deregulation, insulin-like growth factor-2 mRNA-binding proteins (IGF2BPs/IMPs/VICKZs) binding RNA, interleukin-12 signaling, intrinsic pathway for apoptosis, regulation of TP53 expression and degradation, interleukin-12 family signaling, cell cycle, and aberrant regulation of mitotic cell cycle due to RB1 detects” (Supplementary Figure S3B).

We selected high expression and consistency six RBP genes (FUS, HNRNPA2B1, CANX, EIF1, CALR, and POLR2A) for further coexpression analysis (Figures 3(d) and 3(e)). Through analysis, we found eight RASGs (MDM2, GOLGA2P7, NFE2L1, KDM4A, FAM111A, CIRBP, IDH1, and MCM7) that could be regulated by RBP (Figures 3(d) and 3(f)). Read distribution of the two RASGs (FAM111A and CIRBP) is shown in Supplementary Figure S3C. The coexpression regulatory network was conducted to further elucidate the regulatory and interaction relationship between RBPs and AS. Apoptotic progress [33], protein phosphorylation [34], and NF-kappaB cascade [35] revealed by the GO analysis of RASGs regulated by RBPs (Figure 3(d)) were closely related to diabetic retinopathy.

### 3.4. Validation of the RBPs Using mRMVECs and STZ Induced Diabetic Mouse

**Figure 4:**
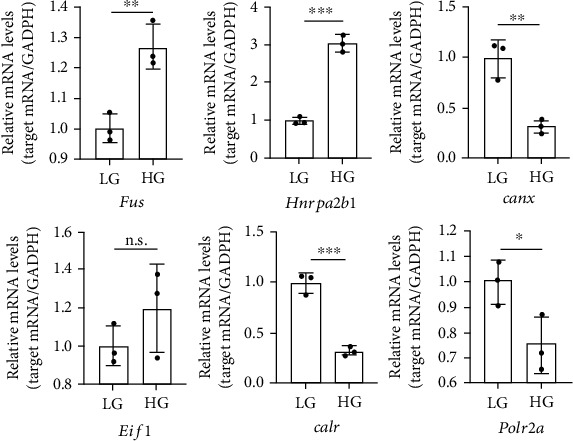
mRMVECs were exposed to hypoglycemia (5.5 mM) or hyperglycemia (25 mM) for 48 h. qRT-PCR was performed to detect differentially expressed 6RBP genes. GAPDH was detected as the internal control. Data are expressed as mean ± SD (*n* = 3, from separate cell samples). ^∗^*p* < 0.05, ^∗∗^*p* < 0.01, and ^∗∗∗^*p* < 0.001, n.s.: not significant, by 2-tailed Student's *t*-test.

**Figure 5:**
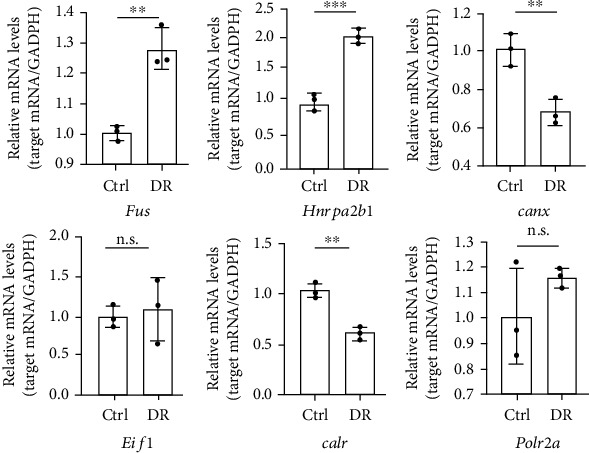
qRT-PCRs were conducted to detect 6 differently expressed genes in mouse retinas (*n* = 3) after diabetes mellitus induction. GAPDH was detected as the internal control. Data are expressed as mean ± SD. ^∗^*p* < 0.05, ^∗∗^*p* < 0.01, and ^∗∗∗^*p* < 0.001, n.s.: not significant, by 2-tailed Student's *t*-test.

Next, we investigated whether six RBP genes (*Fus*, *Hnrnpa2b1*, *Canx*, *Eif1*, *Calr*, and *Polr2a*) were altered upon high glucose (HG) or low glucose (LG) *in vitro* and *in vivo*. mRMVECs were cultured in a medium containing high glucose to mimic the diabetes stress. Compared to LG, HG increased gene expression after 48 h of treatment (Figure 4); *Fus* and *Hnrnpa2b1* were increased while *Canx*, *Calr*, and *Polr2a* were decreased in the HG group compared to those in the LG group (*p* < 0.05). Next, we determined the expression of these six genes in mouse retinas after diabetes mellitus induction. *Fus* and *Hnrnpa2b1* were increased, and *Canx* and *Calr* were decreased in the retinas of diabetic mice compared to nondiabetic mice (*p* < 0.05). However, there was no significant difference in the expression of *Eif1* and *Polr2a* in diabetic mice (Figure 5). The expression of four genes (*Fus*, *Hnrnpa2b1*, *Canx*, and *Calr)* in the retinas of diabetic mice was consistent with cell experiment.

## 4. Discussion

Diabetic retinopathy (DR) is a chronic and serious eye complication associated with diabetes mellitus (DM). Its underlying pathological mechanism is not completely clear. Recent studies have attempted to identify the dysregulation of alternative splicing (AS) associated with the risk of developing this severe diabetic complication. AS regulates gene expression patterns at the posttranscriptional level and expands transcriptomic and proteomic diversity. However, imbalances in the splicing process are affected by an estimated 50% of disease-causing mutations [36]. To the best of our knowledge, only few studies have reported functions of AS in DR [16, 37, 38]. Perrin et al. and Jiang et al. found that alternative splicing of the VEGF gene results in 2 isoforms, the proangiogenic isoforms (VEGFxxxa) and the antiangiogenic isoforms (VEGFxxxb); VEGFxxxb has an antiangiogenic effect and protects DR patients from hyperglycemia through inhibition of proliferation and migration of retinal endothelial cells [37, 38]. Moreover, another study suggested that TUBD1 is the most relevant alternative splicing gene that may be used to differentiate severe DR patients from diabetic patients without DR. The absence of coexpression of TUBD1 a and b (Vab) isoforms is significantly associated with the risk of developing DR, specifically proliferative retinopathy, whereas the coexpression of TUBD1 isoforms a, b, and d (Vabd) has been associated with risk for developing nonproliferative retinopathy [16]. These investigations suggested the role of AS in shaping the susceptibility to DR.

In the present study, we applied bioinformatics methods to identify the DEGs by using previously reported gene expression data from HRECs treated with different doses of glucose. We found that upregulated genes were mainly enriched in the p53 signaling pathway and cell cycle pathways. The downregulated genes were mainly concentrated in amino sugar and nucleotide sugar metabolism, lysosome, etc. These functional pathways were closely related to DR. We further analyzed the AS events in the dataset. Functional enrichment analysis showed that the main functional pathways were the p53 signaling pathway, apoptosis, insulin resistance, etc. Coregulation network of RASGs and RBPs further identified the involvement of gene-level in the pathological changes in DR (Figure 3(d)); FAM111A, CIRBP, GOLGA2P7, MDM2, NFE2L1, KDM4A, MCM7, and IDH1 (Figure 3(f)) were the most relevant RASGs. The abnormal alternative splicing of these genes is directly related to the high glucose treatment of HRECs. *Fam111a* has been found to be downregulated in the retina of STZ-induced rats compared with normal rats [39]. Stress-inducible genes *Cirbp* has been shown to be commonly induced in most types of retinal cells STZ-induced diabetic murine retinas [40]. PI3K inactivation through inactivating AKT/MDM2/p53 signaling pathway prevents vitreous-induced proliferation, migration, and contraction of human retinal pigment epithelium cells [41]. The upregulated novel lncRNA *MSTRG.15047.3* might interact with nearby uplocated CBFTA2T2 in a *cis*-regulatory way, *trans*-regulating cell cycle, and neovascularization via *MCM7* in both diabetic patients' aqueous humor and serum samples, compared with normal controls [42]. Long et al. [43] uncovered that high glucose promotes the secretion of VEGF in diabetic nephropathy by downregulating the promoter of the host *MCM7* gene and downregulating miR-93. These RASGs provide a theoretical basis for our further research investigating the mechanism by which AS modulates DR progression.

AS is a highly dynamic and complicated process, regulated by cis-acting factors, trans-acting factors, transcriptional environment, and core spliceosome [44]. RBP binds cis-elements and trans-acting factors in introns and exons and regulates splice site selection by promoting or repressing definition interactions [45, 46]. Systematic identification of corresponding splicing regulators and their cognate binding sites are essential for a more comprehensive understanding of the function and regulation of alternative splicing networks [47]. Thus, understanding of RBPs, as well as the repertoires of detected splice variants across cellular conditions in the context of normal and disease physiology, is of vital importance to unravel the molecular mechanism of DR.

The functional pathways of RBPs were mainly enriched in mRNA splice site selection, RNA splicing, nuclear RNA splicing, via spliceosome, and so on. The results of the functional analysis indicated that RBPs might have a modulatory role of AS during the progression of DR. The coexpression analysis was conducted to explore the regulatory and interaction relationship between selected RBPs (FUS, HNRNPA2B1, CANX, EIF1, CALR, and POLR2A) and its regulated RASGs (MDM2, GOLGA2P7, NFE2L1, KDM4A, FAM111A, CIRBP, IDH1, and MCM7). Through further functional enrichment analysis, apoptotic progress, protein phosphorylation, and NF-kappaB cascade were closely related to diabetic retinopathy. By searching related literature of these six RBPs, we found that HNRNPA2B1, CALR, EIF1, and POLR2A are associated with DR. The TR-hnRNPA2B1 complex has been reported to repress proliferation, migration, and tube formation of HRECs in hyperglycemia through the STAT-4/miR-223-3p/FBXW7 signaling pathway [48]. *Calr* expression is altered in the retina of rats with diabetic retinopathy compared to healthy control rats [49]. Among PPAR*α*-bound genes, EIF1 is one of the overlapped genes with pemafibrate-induced genes; pemafibrate-treated HUVECs showed a reduced inflammatory reaction and protective effects against diabetic retinopathy [50]. Hebsgaard et al. found a positive control probe POLR2A in proliferative diabetic retinopathy (PDR) patients [51].

Some RBPs, such as FUS and CANX, have not been reported to be associated with DR. FUS is causally associated with oncogenesis and neurodegeneration, and the FUS protein has been identified as a major component of intracellular inclusions in neurodegenerative disorders, including ALS and frontotemporal lobar degeneration [52]. DR is not only a microvascular complication but also a neurodegenerative disease induced by DM [53]. We proposed that FUS may be associated with the disorders of retinal ganglion cells induced by DR. As a chaperone, CANX is closely related to the function of the endoplasmic reticulum (ER) such as ER-phagy and ER-retention [54–56]. A previous study indicated that ER stress causes the damage of retinal blood vessels and neurons in DR [57]. Thus, we assume that CANX may be associated with ER stress induced by high glucose conditions. While the above studies mainly focused on another pathological action of six RBPs, our results revealed that RBP indeed had an important role in the regulation of ASEs. These abnormal RBPs provided more ideas and methods for our follow-up research on the regulation of ASEs.

Finally, we investigated whether six RBP genes (*Fus*, *Hnrnpa2b1*, *Canx*, *Eif1*, *Calr*, and *Polr2a*) were altered upon high glucose or low glucose *in vitro* and *in vivo*. qRT-PCR analysis (Figures 4 and 5) showed that *Fus*, *Hnrnpa2b1*, *Canx*, *Calr*, and *Polr2a* were the significant difference in mRMVECs and *Fus*, *Hnrnpa2b1*, *Canx*, and *Calr* were the significant difference in the diabetic mouse model with DR (two months) compared to control groups. The results indicated that these genes may be triggering factors in diabetes-induced retinal pathogenesis.

A limitation of our study is that we do not conduct further verifications in human specimen to explore the difference of RBP expression. However, many future preclinical studies are needed conduct in animal models. The mechanism of identifying diabetic retinopathy in animal models will help us find potential therapeutic targets. Additional questions emerged from our study that remain to be fully answered, including the regulatory mechanisms of RBP in ASEs which are induced by hyperglycemia and the effect of these mechanisms.

## 5. Conclusions

In summary, this is the first study that elucidated the coexpression networks between RBPs and their regulated ASEs during the development of DR and predicted that RBPs (FUS, HNRNPA2B1, CANX, EIF1, CALR, and POLR2A) were potential targets of DR, which acts on key ASEs (MDM2, GOLGA2P7, NFE2L1, KDM4A, FAM111A, CIRBP, IDH1, and MCM7) to regulate many pathological pathways (apoptotic progress, protein phosphorylation, and NF-kappaB cascade). RBP genes (*Fus*, *Hnrnpa2b1*, *Canx*, and *Calr*), which were differentially expressed in high-glucose-treated mRMVECs and diabetic mouse, may be potentially used as new molecular intervention targets of DR.

## Figures and Tables

**Table 1:** Primers for qRT-PCR assay.

Gene	Primers (5′-3′)
*Fus*	F: TTCAGACAACAATACCATCTTCGTG R: GCTGTCCAGTTTTCTTGTTTGTCTT
*Hnrnpa2b1*	F: CATTGATGGCAGGGTAGTTGAG R: CCTTAATTCCACCAACAAACAGC
*Canx*	F: TAGAATGTGGTGGTGCCTATGTG R: CATCTGGCCTCTTAGCATGTTTT
*Eif1*	F: TATCCTAGCCGGGAGGTGTT R: GCTTGTTCCTTCACATGGCA
*Calr*	F: AATCCTGAATACAAGGGCGAGTG R: GATCTAGGCCCAGTACAGCAAAA
*Polr2a*	F: TTGTATCCGTACCCACAGCA R: CATGATCAGCTCCCCATTCT
*Gadph*	R: CCTCGTCCCGTAGACAAAATG F: TGAGGTCAATGAAGGGGTCGT

## Data Availability

The data used to support the findings of this study are available from the corresponding author upon request.

## Supplementary Materials

Supplementary Materials Figure S1: Analysis of functional pathways of genes differentially expressed in hyperglycemia (HG) and hypoglycemia (LG) in human retinal endothelial cells (HRECs). (A, B) The GO analysis of differentially expressed genes (DEGs), dividing into up- and downregulated genes, respectively. (C, D) The Reactome analysis of DEGs, dividing into up- and downregulated genes, respectively. Figure S2: Transcriptome analysis of alternative splicing events (ASEs) in HRECs treated with LG and HG. (A) Classification of all the detected ASEs. *X*-axis: ASEs number. (B) The KEGG analysis of regulated alternative splicing genes (RASGs) in HG samples compared to LG samples. (C) The KEGG analysis of genes overlapped by DEGs and RASGs. Figure S3: The analysis of differential RNA-binding proteins (RBPs) and RASGs between HG and LG. (A) The KEGG analysis of RBPs regulated alternative splicing events for LG samples and HG samples. (B) The Reactome analysis of RBPs regulated alternative splicing events for LG samples and HG samples. (C) Read distribution of the two of the RASGs.
