# Supplementary material for: RNA-Binding Proteins and Alternative Splicing Genes Are Coregulated in Human Retinal Endothelial Cells Treated with High Glucose
Source: J Diabetes Res. 2022 Mar 9;2022:7680513. doi: 10.1155/2022/7680513 (PMC8926481; doi:10.1155/2022/7680513)
Supplement: Supplementary Materials — Figure S1: Analysis of functional pathways of genes differentially expressed in hyperglycemia (HG) and hypoglycemia (LG) in human retinal endothelial cells (HRECs). (A, B) The GO analysis of differentially expressed genes (DEGs), dividing into up- and downregulated genes, respectively. (C, D) The Reactome analysis of DEGs, dividing into up- and downregulated genes, respectively. Figure S2: Transcriptome analysis of alternative splicing events (ASEs) in HRECs treated with LG and HG. (A) Classification of all the detected ASEs. X-axis: ASEs number. (B) The KEGG analysis of regulated alternative splicing genes (RASGs) in HG samples compared to LG samples. (C) The KEGG analysis of genes overlapped by DEGs and RASGs. Figure S3: The analysis of differential RNA-binding proteins (RBPs) and RASGs between HG and LG. (A) The KEGG analysis of RBPs regulated alternative splicing events for LG samples and HG samples. (B) The Reactome analysis of RBPs regulated alternative splicing events for LG samples and HG samples. (C) Read distribution of the two of the RASGs. [file 7680513.f1.docx]

**Supplementary information**


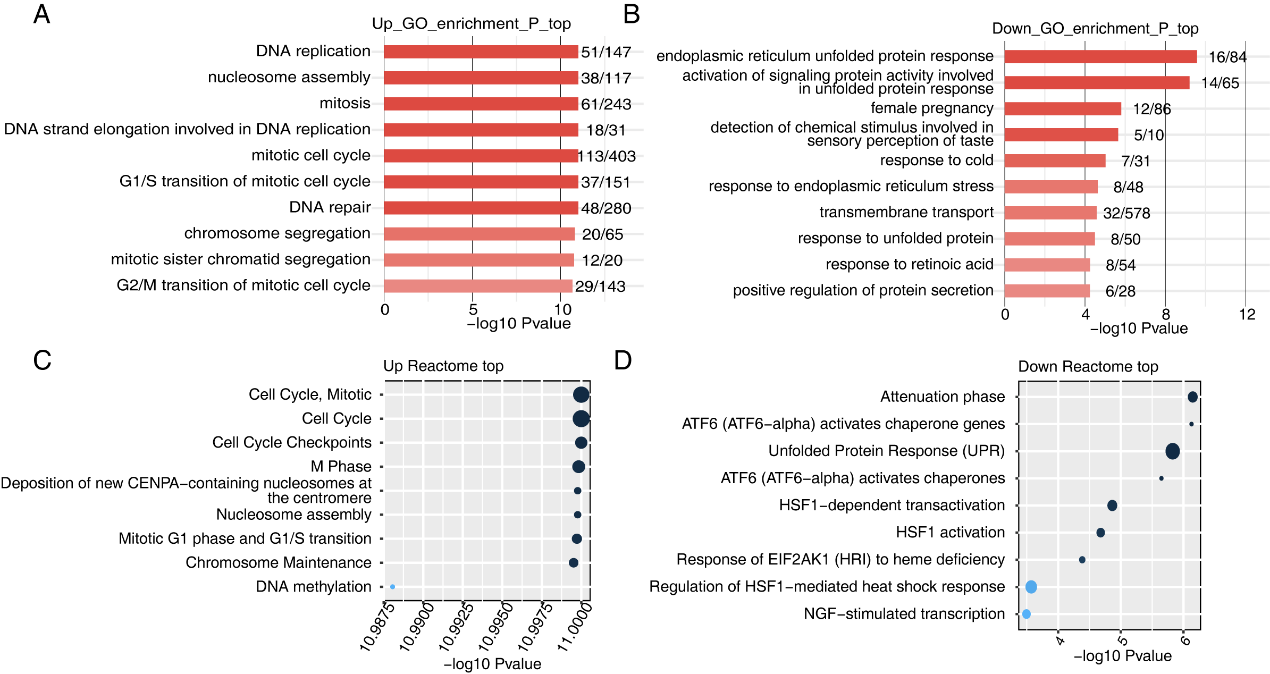


**Figure. S1** Analysis of functional pathways of genes differentially expressed in

hyperglycemia and hypoglycemia in human retinal endothelial cells (hRECs). (**A-B**) The GO analysis of DEGs, dividing into up and down-regulated genes respectively. (**C-D**) The Reactome analysis of DEGs, dividing into up and down-regulated genes respectively.


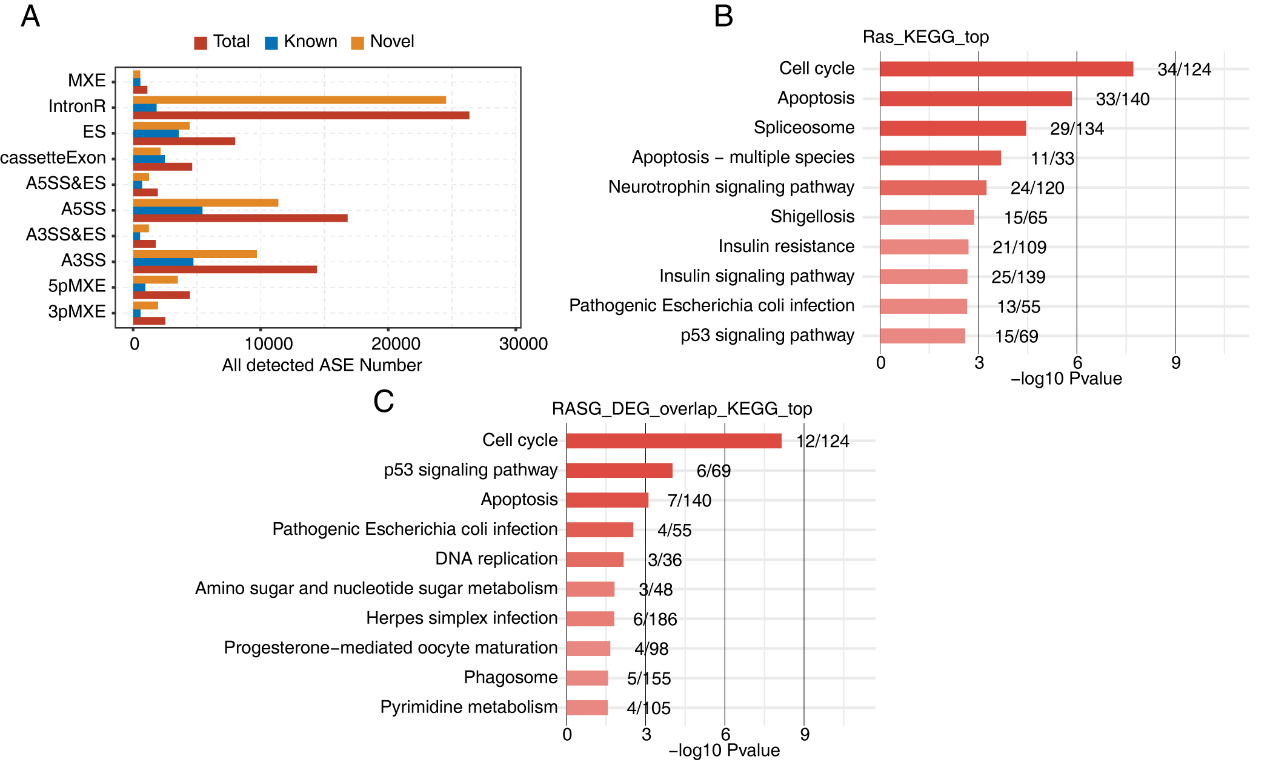


**Figure. S2** Transcriptome analysis of alternative splicing events in human retinal

endothelial cells (hRECs) treated with LG and HG. (**A**) Classification of all the detected AS events (ASEs). X-axis: ASEs number. (**B**) The KEGG analysis of alternative splicing genes (RASGs) in Hyperglycemia samples compared to Hypoglycemia samples. (**C**) The KEGG analysis of genes overlapped by DEGs and RASGs.

**
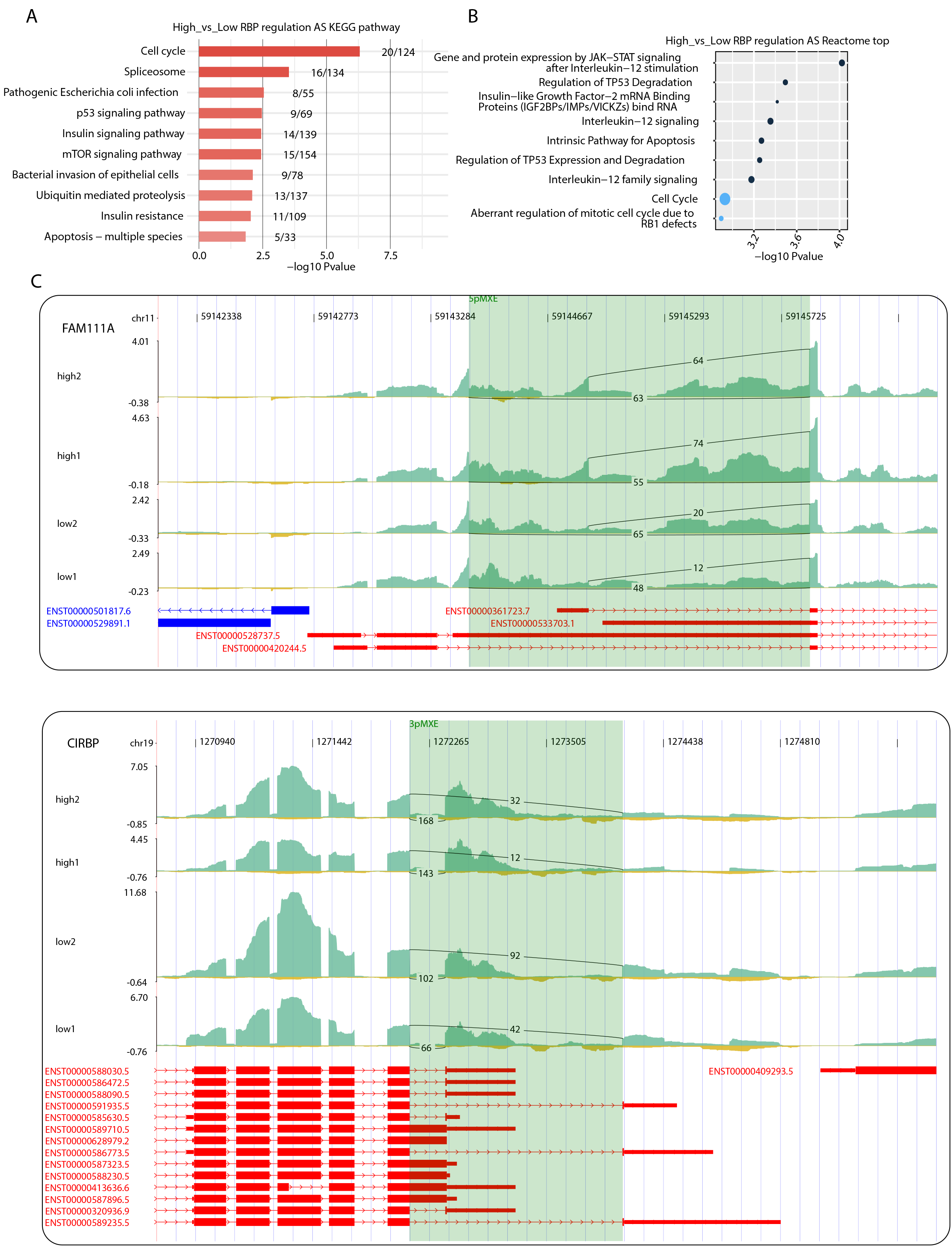
**

**Figure. S3** Differential RNA-binding proteins RASGs analysis between HG and LG. (**A**) The KEGG analysis of RBPs regulated alternative splicing events for hypoglycemia samples and hyperglycemia samples. (**B**) The Reactome analysis of RBPs regulated alternative splicing events for hypoglycemia samples and hyperglycemia samples. (**C**) Reads distribution of the two of the RASGs.
